# Prevalence of *Dirofilaria immitis* infection in dogs in Henan province, central China

**DOI:** 10.1051/parasite/2016054

**Published:** 2016-10-14

**Authors:** Shuai Wang, Nian Zhang, Zhenchao Zhang, Dong Wang, Zhijun Yao, Haizhu Zhang, Jingbo Ma, Bin Zheng, Hongbin Ren, Shiguo Liu

**Affiliations:** 1 Department of Human Parasitology, School of Basic Medical Sciences, Xinxiang Medical University Xinxiang Henan 453003 PR China; 2 Xinxiang Assegal Medical Examination Institute Xinxiang Henan 453003 PR China

**Keywords:** *Dirofilaria immitis*, Prevalence, Dog, Central China

## Abstract

The heartworm *Dirofilaria immitis* is the causative agent of cardiopulmonary dirofilariosis in dogs and cats, and also infects humans. However, there has been no study on dirofilariasis in dogs in central China. From March 2015 to February 2016, sera from 1176 randomly selected household dogs from Henan province, central China were examined for *D. immitis* antigen using the Canine Heartworm Antigen Test Kit. The overall seroprevalence of *D. immitis* in dogs in Henan province was 13% (155/1176). The prevalence was significantly higher in older dogs and dogs kept outdoors, compared to the younger ones and those sheltered indoors. No significant difference of prevalence was observed between sexes. The results suggest that the risk of exposure to *D. immitis* in dogs is high in Henan, and prophylaxis against the parasite is advisable to decrease the incidence of canine dirofilariosis in this region.

## Abstract (fr)

Le Nématode *Dirofilaria immitis* est l’agent causal de la dirofilariose cardiopulmonaire chez les chiens et les chats, et infecte aussi les humains. Cependant, il n’y a pas de rapport sur la dirofilariose chez les chiens dans le centre de la Chine. De mars 2015 à février 2016, les sérums de 1176 chiens domestiques choisis au hasard dans la province du Henan, en Chine centrale, ont été examinés pour l’antigène de *D. immitis* en utilisant le kit Canine Heartworm Antigen Test. La séroprévalence globale de *D. immitis* chez le chien dans la province du Henan était de 13 % (155/1176). La prévalence était significativement plus élevée chez les chiens âgés et les chiens abrités en plein air, par rapport aux plus jeunes et ceux abrités à l’intérieur. Aucune différence significative de prévalence n’a été observée entre les sexes. Les résultats suggèrent que le risque d’exposition à *D. immitis* chez les chiens est élevé dans le Henan, et il est conseillé d’utiliser la prophylaxie contre le parasite pour diminuer l’incidence de la dirofilariose canine dans cette région.

## Introduction

The causative agent of cardiopulmonary dirofilariasis, *Dirofilaria immitis* (heartworm), affects domestic dogs, cats, and various wild mammals, with increasing incidence in temperate and tropical areas [9, 12, 16]. As mosquito-borne zoonotic pathogens, heartworms can also be transmitted to humans, where they cause diseases such as pulmonary dirofilariasis and subcutaneous dirofilariasis [5, 19]. Adult *D. immitis* worms can survive for seven years or more in dogs, usually producing chronic inflammatory vascular disease [19]. In addition, the simultaneous death of groups of adult worms can trigger an acute disorder characterized by the exacerbation of inflammatory reactions and the occurrence of serious thromboembolism that put the life of the infected dogs at immediate risk [14].

There are many reports on the prevalence of *D. immitis* infection in dogs from various countries, including Korea [3], Iran [10], Poland [11], Portugal [1], Costa Rica [18], and Hungary [2]. In recent years, studies of *D. immitis* infection in dogs have been undertaken in different regions of China (Table 1 [6–9, 13, 20–22]). These reports show that canine dirofilariasis is endemic in China. However, there has been no study on dirofilariasis infection in dogs in Henan, central China.

**Table 1.** Prevalence of *Dirofilaria immitis* infection in dogs in China.

Localities	Year of sampling^A^	No. tested	Positive (%)	Method^B^	Reference
Taipei	1998–1999	664	89 (13.4)	ELISA	[6]
Taiwan	1993–1997	2065	803 (38.9)	Necropsy and microscopic examination	[22]
Heilongjiang	1996–2004	178	2 (1.1)	Necropsy	[21]
Changchun	<2007	62	32 (51.6)	ELISA and microscopic examination	[8]
Guizhou	<2010	300	15 (5.0)	Immunochromatographic strip	[7]
Dandong	2003–2010	886	213 (24.0)	PCR and microscopic examination	[9]
Kunming	2010–2011	30	2 (6.7)	ELISA	[20]
Chongqing	2010–2011	30	3 (10.0)	ELISA	[20]
Nanchang	2010–2011	30	3 (10.0)	ELISA	[20]
Fuzhou	2010–2011	50	6 (12.0)	ELISA	[20]
Guangzhou	2010–2011	60	9 (15.0)	ELISA	[20]
Shenzhen	2010–2011	80	15 (18.8)	ELISA	[20]
Nanning	2010–2011	30	4 (13.3)	ELISA	[20]
Shenyang	2009–2012	528	67 (12.7)	ELISA	[13]

A Years of sampling are listed as published in the references. In cases where this information was not available, the year listed here is the year when the study was published, as indicated by “<”.

B ELISA: enzyme-linked immunosorbent assay; PCR: polymerase chain reaction.

Adult worms of *D. immitis* reside in pulmonary arteries and the right ventricles, resulting in production of blood-circulating microfilariae in dogs as natural hosts [12]. Because dogs with a low worm burden are usually asymptomatic, primary diagnostic screening by detecting blood microfilariae or circulating heartworm antigens is necessary prior to treatment [17]. However, due to occult infection in some cases, antigen testing is considered the most sensitive diagnostic method [17]. Therefore, in the present study, we studied the seroprevalence of *D. immitis* infection in domestic dogs in central China for the first time and evaluated the main risk factors associated with exposure to *D. immitis* in this area.

## Materials and methods

### Ethical statement

The study was reviewed and approved by the Ethics Review Committee of the Xinxiang Medical University (Reference No. 2015016).

### Study site

**Figure 1.**
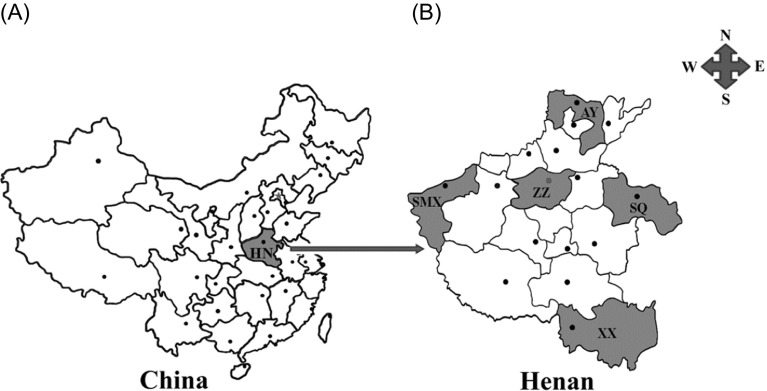
Geographic distribution of the sampling sites in Henan province, China used in this study. A: Henan province (HN, shadowed areas) is located in the central part of the mainland China. B: Shadowed areas are the sampling locations for the present survey. AY: Anyang; SMX: Sanmenxia; ZZ: Zhengzhou; XX: Xinyang; SQ: Shangqiu.

The study was conducted in Henan province, located in the central part of mainland China (Fig. 1), and covering an area of 167,000 km^2^ and a population of approximately 106.01 million. Its geographical position is at east longitude 110°21′–116°39′ and at north latitude 31°23′–36°22′. The Yellow River passes through central Henan. The area has a continental monsoon climate, with four distinctive seasons. The average annual temperature is 12.1–15.7 °C, with a mean annual rainfall of 532.5–1380.6 mm. As shown in Figure 1, there are 17 provincial cities distributed in Henan province, with the city of Zhengzhou as its capital. Five cities including Anyang (35°13′–36°22′ N, 113°37′–114°58′ E), Sanmenxia (33°31′–35°05′ N, 110°21′–112°01′ E), Zhengzhou (34°16′–34°58′ N, 112°42′–114°13′ E), Xinyang (31°46′–31°52′ N, 114°01′–114°06′ E), and Shangqiu (33°43′–34°52′ N, 114°49′–116°39′ E), located in the northern, western, central, southern, and eastern parts of Henan province, were selected for sample collection.

### Sample collection

A total of 1176 blood samples of domestic dogs were collected from these five cities in Henan province between March 2015 and February 2016. Dog owners were asked for details of the animals’ age, sex, rearing condition, and medical history using a structured questionnaire. Blood samples were centrifuged and sera were recovered and transferred to 1.5 mL Eppendorf tubes. All sera were then stored at −80 °C until testing for circulating *D. immitis* antigens.

### Test for *D. immitis* antigens

In order to identify *D. immitis* infection, all serum samples were analyzed for the *D. immitis* antigens using the Canine Heartworm Antigen Test Kit (IDEXX Laboratories, Westbrook, ME, USA) according to the manufacturer’s instructions. When testing samples from dogs with more than two adult female worms, sensitivities and specificities of this kit are 94% and 98% for *D. immitis*, respectively [4].

### Statistical analysis

Statistical analysis was performed using SPSS 20 software for Windows (SPSS Inc, Chicago, IL, USA). Statistical analyses of *D. immitis* prevalence in different variables were performed by the χ^2^-test. The differences were considered statistically significant if *p* < 0.05.

## Results and discussion

As shown in Table 2, the overall recorded seroprevalence of *D. immitis* in dogs in Henan province, central China was 13.18% (155/1176). Compared with other provinces or cities in China, it was lower than the values of 51.6% in dogs in a study performed in Changchun [8], 38.9% in Taiwan [22], 24.0% in Dandong [9], and 18.8% in Shenzhen [20], similar to those observed in Shenyang (12.7%) [13] and Nanning (13.3%) [20], but higher than those observed in Heilongjiang (1.1%) [21], Guizhou (5.0%) [7], Kunming (6.7%), Chongqing (10.0%), Nanchang (10.0%), and Fuzhou (12.0%) [20]. The differences in prevalence of *D. immitis* among these regions may be due to differences in ecological and geographical factors, detection methods used, survey periods, sample sizes, and breed of dog populations in these areas. In the present study, the seroprevalence of *D. immitis* in males was 14.26% (88/617) and in females 11.99% (67/559) (Table 2). Although the seroprevalence in males was higher than in females, the difference was not significant (*p* > 0.05). This is in agreement with previous studies [9, 13].

**Table 2.** Seroprevalence of *Dirofilaria immitis* infection in dogs in Henan province, central China.

Variable	No. examined	No. positive	Prevalence (%)
Region			
Anyang	224	40	17.86^bc^
Sanmenxia	235	23	9.79^a^
Zhengzhou	256	19	7.42^a^
Xinyang	242	28	11.57^ab^
Shangqiu	219	45	20.55^c^
Sex			
Male	617	88	14.26
Female	559	67	11.99
Rearing condition			
Indoor	545	52	9.54^a^
Outdoor	631	103	16.32^b^
Age (years)			
≤3	318	22	6.92^a^
3 ~ 6	573	70	12.22^b^
≥ 6	285	63	22.11^c^
Total	1176	155	13.18

Values bearing a different superscript letter (a–c) within a column differ significantly from one another (*p* < 0.05).

A significant difference was observed in prevalence of *D. immitis* between dogs sheltered in different conditions [9, 13]. In this study, the seroprevalence of *D. immitis* infection in outdoor dogs (16.32%) was significantly higher than that in indoor dogs (9.54%; *p* < 0.01). A similar higher prevalence was also observed in outdoor dogs by Hou et al. [9] and Liu et al. [13]. The possible reason is that dogs outdoors had a greater chance of being bitten by mosquitoes [9, 13]. In the present study, the highest prevalence of infection (22.11%) was detected in six-year-old or older dogs, followed by intermediate prevalence (12.22%) in the 3–6 year age group, while the prevalence found in dogs in the ≤3 year age group was 6.92% (Table 2). The prevalence of *D. immitis* infection in dogs increased significantly (*p* < 0.05) with the increase in age. The difference in prevalence with respect to age coincides with other studies where higher prevalence was observed in the adult group than in the juvenile one [10, 13, 15]. These findings suggest that the risk of exposure to *D. immitis* increases with age.

## Conclusions

In conclusion, a high prevalence of *D. immitis* infection (13%) was found in domestic dogs in Henan, central China. Therefore, lifelong chemoprophylaxis is needed to prevent canine dirofilariosis. Monitoring the prevalence of this particular nematode among domesticated dogs is also important because it poses a serious health risk to humans.

## Conflict of interest

The authors declare that they have no conflict of interest.
